# Timing of Immune Escape Linked to Success or Failure of Vaccination

**DOI:** 10.1371/journal.pone.0012774

**Published:** 2010-09-16

**Authors:** Jeanette C. Reece, Liyen Loh, Sheilajen Alcantara, Caroline S. Fernandez, John Stambas, Amy Sexton, Robert De Rose, Janka Petravic, Miles P. Davenport, Stephen J. Kent

**Affiliations:** 1 Department of Microbiology and Immunology, University of Melbourne, Melbourne, Australia; 2 Centre for Vascular Research, University of New South Wales, Sydney, Australia; New York University, United States of America

## Abstract

Successful vaccination against HIV should limit viral replication sufficiently to prevent the emergence of viral immune escape mutations. Broadly directed immunity is likely to be required to limit opportunities for immune escape variants to flourish. We studied the emergence of an SIV Gag cytotoxic T cell immune escape variant in pigtail macaques expressing the Mane-A*10 MHC I allele using a quantitative RT-PCR to measure viral loads of escape and wild type variants. Animals receiving whole Gag expressing vaccines completely controlled an SIV_mac251_ challenge, had broader CTL responses and exhibited minimal CTL escape. In contrast, animals vaccinated with only a single CTL epitope and challenged with the same SIV_mac251_ stock had high levels of viral replication and rapid CTL escape. Unvaccinated naïve animals exhibited a slower emergence of immune escape variants. Thus narrowly directed vaccination against a single epitope resulted in rapid immune escape and viral levels equivalent to that of naïve unvaccinated animals. These results emphasize the importance of inducing broadly directed HIV-specific immunity that effectively quashes early viral replication and limits the generation of immune escape variants. This has important implications for the selection of HIV vaccines for expanded human trials.

## Introduction

The HIV pandemic continues unchecked and a vaccine is urgently needed. The lack of successful vaccination strategies contrasts starkly with the success of antiretroviral drug therapy. Combinations of drugs that successfully limit HIV replication do not select for drug resistant variants and result in long-term control of infection and near-normal life expectancy. Uniform testing and anti-retroviral treatment is predicted in some models to ultimately control the HIV epidemic [1]. How best to emulate such control of HIV with immune responses induced by vaccination remains unclear [2].

Many immune escape mutations (EM), particularly those in conserved proteins such as Gag, are likely to inflict at least some reduction in viral replication capacity – known as a “fitness cost”. This is demonstrated clearly by reversion of mutations back to the fitter wild type (WT) sequences upon transmission to new hosts not able to mount the same immune response [3], [4], [5]. The transmission of HIV strains with multiple CD8+ cytotoxic T lymphocyte (CTL) immune escape mutations in the Gag protein results in lower levels of viremia in recipients [6], [7]. Most immune escape mutations take some time to revert in the new hosts; this is an advantage to the new host, as the less fit escape variants dominate during acute infection, a time when considerable loss of CD4 T cells can occur [8], [9].

Immune escape can occur at various times after natural infection – escape variants to strains that induce early CTL responses will tend to appear earlier, sometimes during acute infection [10]. Weaker, subdominant or later arising immune responses will tend to result in delayed and slower escape [11]. The rate at which immune escape proceeds is dependent in part on the availability of receptive target CD4 T cells [12]. Target cells typically decrease over time, slowing down escape from later-arising immune responses.

The prior generation of effective immune responses through vaccination could effectively quash viral replication and limit opportunities to escape. However, we recently postulated that if replication is sufficiently high, pressure from vaccine-induced response might force immune escape variants to arise early (earlier than they might have during natural infection), reducing the flexibility of the overall immune response [13]. This would be particularly counter-productive if a highly dominant immune response (to a now escaped epitope) subverted effective but subdominant immune responses [14].

## Results

We studied immune escape in 22 pigtail macaques (*Macaca nemestrina*) involved in previously published SIV vaccine experiments. Macaques were either vaccinated with regimens that either expressed (a) whole SIV Gag protein [15], [16], (b) or only the KP9 Gag epitope [17] or (c) were unvaccinated. All macaques studied expressed the Mane-A*10 MHC I allele (recently renamed Mane-A1*08401 [18]) which presents an immunodominant CTL epitope, Gag_164-172_ known as KP9 [19]. This KP9 epitope and typically undergoes escape at the second amino acid (K165R mutant) following challenge with SIV_mac251_ [3]. We can study escape at this epitope in detail on serial plasma SIV RNA samples with quantitative real time PCR (qRT-PCR) assays specific for WT virus or the K165R escape mutant (EM) virus [20]. This qRT-PCR assay provides a “viral load” for both WT and EM variants. We applied this assay to a series of Mane-A*10+ pigtail macaques receiving either Gag or KP9-expressing vaccines to assess the effect of vaccine-induced immunity on the emergence of immune escape and the outcome of SIV infection.

The 22 pigtail macaques involved in published vaccine studies were analyzed as shown in Table 1. All were challenged intravenously with the same stock of SIV_mac251_. Seventeen animals did not receive SIV-expressing vaccines; 13 of these animals were followed for 3 weeks (after which they were placed on ART and entered a therapeutic vaccine protocol) and 4 animals were followed for >20 weeks post infection. Two animals (1335, 2374) received recombinant viral vectors (influenza A viruses) expressing only the KP9 Gag epitope [17]. Three animals received recombinant viral vectors expressing SIV_mac239_ Gag-Pol - two animals (5821, 5827) received a prime/boost regimen with recombinant vaccinia and fowlpox viruses [16] and one animal (5612) received recombinant Kunjin viruses [15]. All the Mane-A*10+ animals from within these vaccine studies were analyzed. The viral loads, CD4 T cell levels and KP9 specific CD8 T cell responses from these studies have previously been reported, and the immune escape profiles from 3 of the naïve animals and the 2 KP9-only vaccinated animals have also been reported. Here we analyze immune escape in all animals and compare the outcomes of infection and timing of escape in these different vaccination scenarios.

**Table 1:** Mane-A*10+ pigtail macaques studied for CTL escape.

Group (reference)	Animal Number	Prior SIV Vaccines (weeks administered)	Timing of SIV_mac251_ infection	KP9 CTL epitope sequence after infection
**KP9 Gag epitope vaccinees** [17]	1335	Influenza–KP9(week 0, 4, 44, 48)	week 54	K**R**FGAEVVP
	2374			K**R**FGAEVVP
**Whole Gag vaccinees** [15], [16]	5821	VV/FPV–SIV(week 0, 4)	week 18	KKFGAEVVP
	5827			KKFGAEVVP
	5612	Kunjin–SIV(week 0, 4, 8, 18)	week 24	K**R**FGAEVVP
**Naïve** (chronic infection) [15], [21]	5284	None	N/A	K**R**FGAEVVP
	5424			K**R**FGAEVVP
	5715			K**R**FGAEVVP
	3C7D			KKFGAEVV**S** *
**Naïve** (followed through acute infection only – placed on ART at week 3) [25]	8014	None	N/A	K**R**FGAEVVP
	9017			K**R**FGAEVVP
	9176			K**R**FGAEVVP
	9183			KKFGAEVVP
	8020			KKFGAEVVP
	8241			KKFGAEVV**S** *
	8244			K**R**FGAEVVP
	8454			KKFGAEVVP
	1.3731			K**R**FGAEVVP
	8240			K**R**FGAEVVP
	9020			KKFGAEVVP
	9021			KKFGAEVVP
	9175			K**R**FGAEVVP

*The P172S CTL escape mutation is not detected by the qRT-PCR assay and these animals were not studied for escape rates.

### Outcomes of SIV challenge

**Figure 1:**
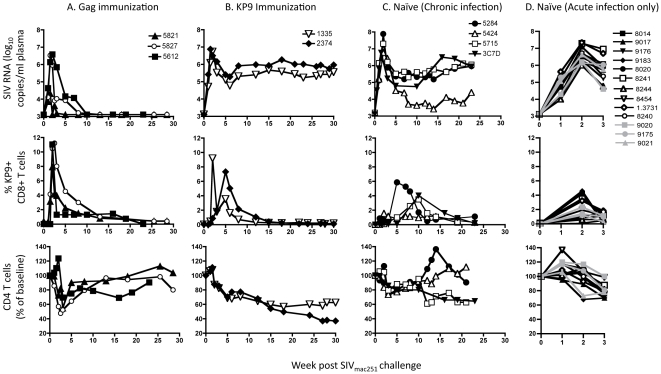
Virologic and immunologic characteristics. Groups of animals were studied for SIV RNA levels in plasma, SIV Gag KP9-specific CD8+ T cell levels by MHC tetramer analyses and CD4 T cell levels in peripheral blood after the same SIV_mac251_ challenge. Animals received either **A.** Vaccines expressing whole SIV Gag, **B.** Vaccines only expressing the SIV Gag KP9 epitope **C.** Naïve unimmunized animals followed for >20 weeks or **D.** Naïve unimmunized animals followed for 3 weeks.

Expression of the Mane-A*10 MHC class I allele by pigtail macaques is associated with reduced viral load and reduced disease compared to non-Mane-A*10+ pigtail macaques [21], [22]. Early and complete control of SIV_mac251_ viremia was observed in 3 Mane-A*10+ pigtail macaques vaccinated with recombinant viral vectors expressing whole SIV Gag (Figure 1A) [15], [16], [23]. In all 3 animals, SIV_mac251_ challenge resulted in detection of SIV RNA for 3-7 weeks at peak levels of 4.3 – 6.5 log_10_ copies/ml prior to control to undetectable levels of SIV RNA using a standard viral load assay. CD4+ T cell levels in peripheral blood dipped to 50-70% of baseline levels during this period of acute viremia, before returning to normal levels. There was a marked expansion of KP9-specific CD8 T cells in the blood, identified using a Mane-A*10/KP9 tetramer as previously described [24]. KP9-specific CD8 T cells peaked at 8-12% shortly after acute infection. Taken together, these animals represent a successful outcome of T cell based vaccination.

In contrast, a dismal outcome was observed following SIV_mac251_ challenge of animals immunized with recombinant influenza A vectors expressing only the 9 amino acid KP9 SIV Gag CD8 T cell epitope alone [17]. Levels of viremia after acute infection were sustained at 5–6 log_10_ copies/ml, approximately 1000-fold higher than in the successfully vaccinated animals described above (Figure 1B). A gradual and sustained loss of CD4+ T lymphocytes resulted in both animals. This progressive SIV infection occurred despite the marked expansion of SIV Gag KP9 specific CD8 T cells, peaking early at 7–9% following infection.

Given these dichotomous outcomes, we then analyzed 17 naïve Mane-A*10+ pigtail macaques infected with SIV_mac251_. All 17 animals had high peak viral loads and the 4 animals followed through chronic infection maintained moderate to high viral loads during chronic infection (4–6 log_10_ copies/ml, Figure 1C). All animals showed a decline in CD4 T cells during acute infection – this decline was not different to the vaccinated animals that also had significant acute plasma viremia levels. Two of the 4 animals followed through chronic infection experienced a sustained loss of CD4 T cells. These animals were unvaccinated, and the peak expansion of SIV Gag KP9-specific CD8 T cells in blood was consistently slower (week 5 or later) and lower (1.5–6%) compared to the 5 vaccinated animals above.

### Generation of immune escape

The success of the vaccination strategy using whole Gag-expressing vectors resulted in limited generation of immune escape at the KP9 Gag CD8 T cell epitope. We first sequenced virus to ensure that the canonical K165R escape mutant was the dominant mutation in the animals under study – this was indeed the case for all but 2 or the 17 control animals (3C7D and 8241) which had the less common escape variant P172S (Table 1).

**Figure 2:**
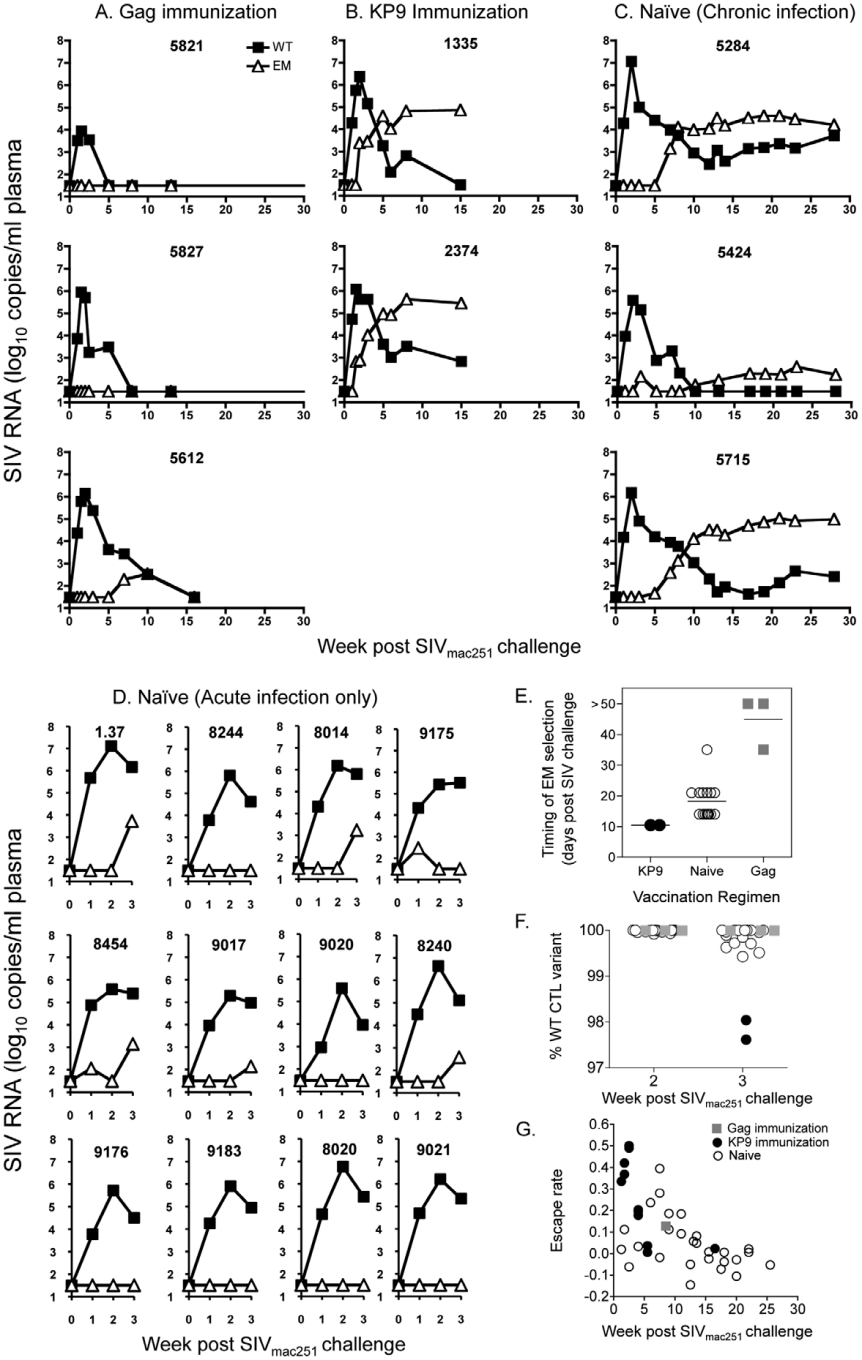
Immune escape at the KP9 epitope. Pigtail macaques in the three vaccine groups (**A.** whole Gag immunized, **B.** KP9 immunized **C.** Naïve unimmunized animals followed for >20 weeks or **D.** Naïve unimmunized followed for 3 weeks) were studied for viral loads of either wild type virus or virus having the K165R escape mutation using a quantitative real time PCR with primers specific for wild type or escape mutant virus. Serial plasma samples were studied after SIV_mac251_ infection. **E.** Minimum estimate of the timing of the start of selection for the K165R EM after SIV infection according to vaccine modality. Each dot represents an individual animal studied. Two animals vaccinated with whole Gag expressing vaccine vectors showed no immune escape and the timing is represented as >50 days. **F.** Proportion of WT virus compared to EM virus at week 2 and 3 after SIV infection. Vaccine groups are shown by different symbols. Each dot represents an individual animal studied. **G.** Rates of escape plotted against time after SIV infection. Each dot represents one animal studied at one time point.

We then employed the qRT-PCR assay on animals with K165R escape to generate serial viral load data for both WT and the K165R KP9 EM variant. In the animals vaccinated whole Gag-expressing vectors, the immune escape variant was not detected at all 2 of 3 animals (Figure 2A). In one animal, the EM variant was detected at low levels (<2.7 log_10_ copies/ml) at weeks 7 and 10 after challenge before disappearing.

The failed vaccination approach based on a single epitope (Gag KP9) predictably resulted in the emergence of a CTL escape variant, as previously reported [17]. The generation and outgrowth of the EM virus was remarkably early and rapid, with the EM variant observed by day 7–10 after inoculation of these animals. The EM variant exceeded WT virus levels within 5 weeks of SIV infection (Figure 2B).

A delayed pattern of CTL escape at the KP9 epitope is observed in the unvaccinated SIV-infected animals. Immune escape was substantially delayed compared to animals vaccinated against KP9 alone, with significant levels of EM virus not appearing until week 5 of infection (3–4 weeks later than the KP9 alone vaccinated animals) and not exceeding WT virus until 7–10 weeks after infection in the 3 animals with chronic SIV and the K165R mutation (Figure 2C, D).

We compared the estimate of the timing of escape starting across the vaccination groups (Fig 2E). Immune escape at the KP9 CTL epitope emerges significantly earlier in the KP9 vaccinated animals than in naïve animals (Wilcoxon signed rank test, p = 0.0005, Fig 2E). We also directly compared the proportion of EM and WT virus during early infection (at week 2 and 3). At week 2, the relative levels of EM and WT virus are similar across the groups, but by week 3 the levels diverge, with more EM virus in the KP9-only vaccines (1-way ANOVA p = 0.0207, Fig 2F).

We calculated the rates of immune escape across all animals and timepoints as previously described [12] (Figure 2G). The selective advantage of escape mutations is higher during early infection and wanes later as a result of the decline in both CD8 T cell numbers and function and changes in CD4 T cell target cells [12]. Indeed, the rates of immune escape were significantly negatively correlated with time post infection (Figure 2G; for all animals *r* = −0.6390, *p*<0.0001; for KP9-vaccinated animals *r* = −0.749, *p* = 0.0149; and for naïve animals *r* = −0.523, *p* = 0.0018. Spearman correlation).

### Breadth of immunity after challenge

**Figure 3:**
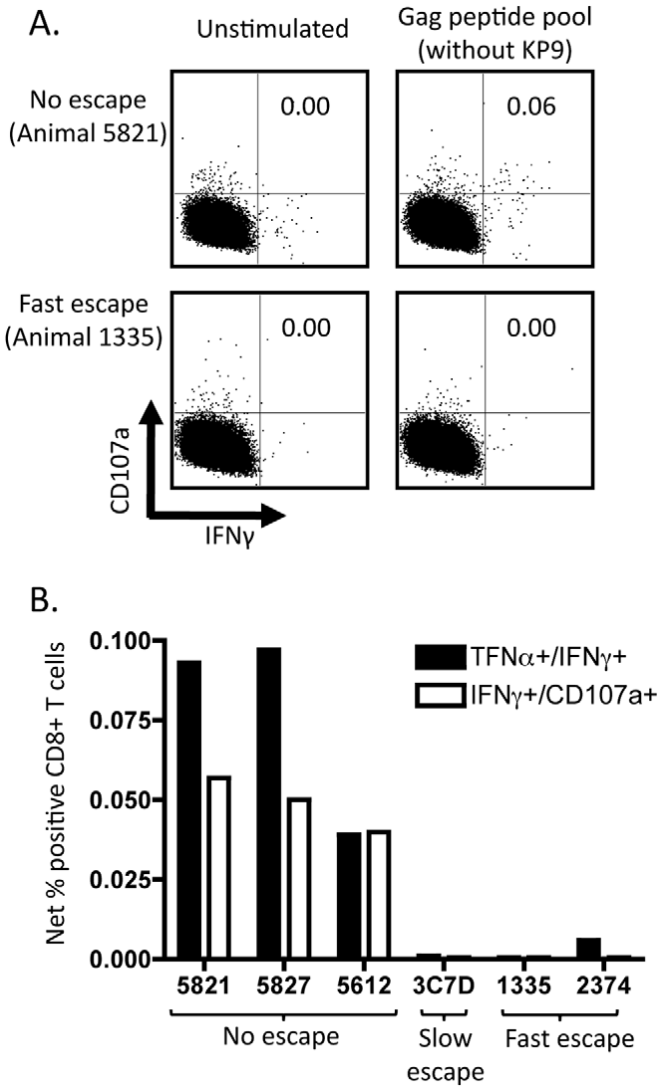
Non–KP9 Gag-specific CD8 T cell immunity. Frozen PBMCs were studied for Gag-specific CD8 T cells expressing TNF-alpha, IFN-gamma and CD107a five weeks after SIVmac_251_ infection. Panel **A** shows examples of an animal receiving whole Gag immunization compared to an animal receiving only KP9 epitope immunization. The percentage of T cells expressing both IFN-gamma and CD107a is shown in the upper right quadrant. Panel **B** shows all animals studied for both net combined TNF-alpha and IFN-gamma as well as combined CD107a and IFN-gamma expression. Sufficient PBMCs were available from only one animal in the group not immunized with SIV-expressing vaccines – this animal received Kunjin vaccines not expressing SIV antigens [15].

The control of viremia and prevention of immune escape at the KP9 CD8 T cell epitope by whole Gag-expressing vaccines suggested that immunity to other regions within Gag may have been generated. Indeed Gag-specific T cell immunity is linked to control of viremia both in pigtail macaques infected with SIV_mac251_ [25] and humans infected with HIV-1 [26]. We therefore went back to study non-KP9 Gag-specific CD8 T cell responses in these animals. We constructed an overlapping Gag 15mer peptide pool that excluded the peptide containing the KP9 epitope sequence and stimulated thawed PBMC (frozen 5 weeks after SIV challenge) to analyze the expression of intracellular TNF-alpha, IFN-gamma and the degranulation marker CD107a as previously described [27]. Detectable non-KP9 Gag-specific CD8 T cell responses were found in the animals with minimal escape at KP9 and control of viremia, but we did not detect responses in the animals exhibiting fast escape and uncontrolled viremia (Figure 3).

## Discussion

HIV-infected subjects who spontaneously control viremia are likely to mount effective combinations of immune responses. Understanding and inducing such effective immunity by vaccination is a priority. We found that inducing CTL responses to a single epitope resulted in the early selection of immune escape species and no control of viremia, consistent with previous macaque vaccine studies [28], [29]. Inducing immune responses to the whole Gag protein was associated with rapid control of viremia and abrogated or limited recrudescent viremia caused by immune escape variants. These data support the importance of a broader base of CD8 T cell responses in facilitating control of SIV viremia by vaccination. We speculate that if vaccination prevents early escape at immunodominant epitopes, this may lead to sustained virus control. The whole Gag immunization strategy could hinder the emergence of the K165R CTL escape mutation in our model by 3 potential mechanisms: it considerably lowers overall viral loads, decreasing the probability of the mutation; this probability is further lowered by the need for multiple simultaneous mutations for the escape from the more diverse CD8 response; and the mutations at multiple epitopes have higher chances of being deleterious or having huge fitness costs for the EM, making the KP9 EM subpopulation weaker [13].

The use of mono-epitope vaccine strategies is likely to be particularly damaging: the recall response following viral challenge will be primarily directed towards the escaping epitope. Subdominant responses, which could otherwise help facilitate viral control [14], are suppressed in this scenario and the progression of infection is similar to that of control animals. This work is of particular relevance to future vaccine approaches as the recent unsuccessful Adenovirus type 5 vector HIV vaccine efficacy clinical trial induced responses on average to only one Gag-specific CD8 T cell epitope per subject [30], [31]. This work predicts that analyses of the presence and rate of CTL immune escape in this clinical phase III trial should reveal a more rapid evolution of escape variants in vaccine recipients compared to unvaccinated controls expressing the same restricting MHC allele.

Several future studies should assist in refining this model of CTL-based control of primate lentiviruses. Although we studied 22 macaques in total, only smaller subsets of these were vaccinated and larger studies will help validate this work. Further, although all macaques expressed the Mane-A*10 MHC I allele which restricts the dominant KP9 Gag response, alternative MHC alleles restricting other useful T cell responses in this outbred group could confound our data. More extensive MHC typing found none of the vaccinated macaques expressed the Mane-B*10 or A*17 alleles previously shown to present the dominant KW9 or AF9 Gag epitopes [11], as yet unknown CTL epitopes could also facilitate viral control.

An additional area requiring further work is that different vaccine vectors will likely play an important role in the immune responses generated. Although we observed a similar magnitude of the KP9 responses after challenge despite different vaccine vectors studied, we did not examine the many other aspects of the quality of the CD8 T cell responses generated by vaccination (e.g cytokine expression [32], ability to rapidly kill infected cells [16], [33], TCR repertoire [34]). These may also impact upon the success of vaccination and escape rates. Other immune responses induced by the whole Gag-expressing vaccines (e.g. CD4 T cell responses) could also play a role in the protective immunity observed, either by direct effector mechanisms or by enhancing CD8 T cell function [35]. Future studies could directly assess the impact of 1, 2, 3 or more CD8 and/or CD4 T cell epitopes expressed by the same vaccine construct. Recently, we have constructed and tested recombinant influenza vectors expressing 3 separate SIV CTL epitopes, all restricted by Mane-A*10 [17], [36], and can now begin to formally address the specific breadth of CTL responses required for adequate viral control in this model.

In summary, our data suggests that inducing broader Gag-specific CD8 T cell immunity by vaccination will be associated with more limited opportunities for viral escape. Inducing responses to single immunodominant CD8 T cell epitopes could be especially damaging when escape ensues, as subdominant responses to the infection may be suppressed. This data has implications for choosing HIV vaccine regimens to progress towards efficacy studies.

## Methods

### Ethics Statement

Experiments on the macaques were approved by the University of Melbourne and CSIRO livestock industries Animal Ethics Committees, approval numbers 1065, 1139, 1164 and 1222. All animals were cared for by dedicated trained animal technicians and humanely euthanised before SIV disease occurred. Animals were held according to Australian NHMRC animal welfare guidelines in group housing with cages 2.0 m high, 3.0 m long and 1.2 m wide. An environmental enrichment strategy for animals provided toys, treats, fresh fruits and vegetables. Specifically, there were regular changes of equipment and toys, facilities to allow swinging and jumping and engaging in activities from floor to ceiling, wood chip substrates on the floor to provide opportunity for foraging, the use of puzzle feeders, placement of food in different locations in the enclosures and access to partially partitioned areas within the enclosures for rest and privacy. There was also access to mirrors and water pools. Animals were monitored daily for health and welfare. All procedures were carried out with sedated animals.

### Animals, Vaccinations and SIV challenge

We studied 22 Mane-A*10+ pigtail macaques (*Macaca nemestrina*) as shown in Table 1. Experiments on the macaques were approved by the University of Melbourne and CSIRO livestock industries Animal Ethics Committees. Pigtail macaques were MHC typed for Mane-A*10 by reference strand-mediated conformational analysis and sequence-specific primer PCR as described [19], [37]. All were challenged with the same stock of SIV_mac251_ IV 40 TCID50. Four animals did not receive any SIV-expressing vaccines. Two animals (1335, 2374) received recombinant influenza A viruses expressing only the KP9 Gag epitope [17]. Two animals (5821, 5827) received recombinant vaccinia and fowlpoxviruses expressing SIV_mac239_ Gag-Pol [16]. One animal (5612) received recombinant Kunjin viruses expressing SIV_mac239_ Gag-Pol [15]. All Mane-A*10+ animals from within these vaccine studies were analyzed.

KP9-specific immune responses were studied on serial blood samples using a Mane-A*10/KP9 tetramer and flow cytometry as previously described [24]. Total SIV RNA levels (viral load) in plasma was studied by real-time PCR as previously described [38]. CD4 T cell depletion in peripheral blood was analyzed by flow cytometry and normalized to baseline levels as previously described [39].

### qRT-PCR for immune escape

To quantify virus levels of WT or EM quasispecies at the KP9 epitope we employed a discriminatory real-time PCR assay as described [20], [40]. Briefly, the assay uses a forward primer specific for either wild-type sequence or specific for the nucleotide mutation encoding the dominant K165R KP9 escape mutant. At each timepoint after infection 10 µl of plasma RNA was to reverse-transcribed and then amplified by qRT-PCR using either WT or EM forward primers. A common reverse primer and FAM-labelled DNA probe were also added for quantification against the appropriate SIV Gag epitope RNA standards using an Eppendorf Realplex^4^ cycler. Analysis was performed using Eppendorf Realplex software. Baselines were set 2 cycles earlier than real reported fluorescence and threshold value was determined by setting threshold bar within the linear data phase. Samples amplifying after 40 cycles were regarded as negative, and corresponded to <1.5-Log_10_ SIV RNA copies/ml of plasma. Analyses of the rate of immune escape were performed as previously described by calculating the change in the viral species over time [12].

### Analysis of breadth of immunity

To analyze the impact of breadth of T cell immunity to Gag (i.e. other than KP9-specific responses), we performed intracellular cytokine staining assays for T cell activation using 5 pools of 25 overlapping 15mers peptides spanning the entire SIV Gag protein as previously described [27]. Briefly, PBMC were stimulated for 6 hrs with the peptide pools (1 µg/ml) and then stained for T cell surface markers (CD3, CD4, CD8), the degranulation marker CD107a and intracellular IFN-gamma and TNF-alpha. The proportion of CD4+CD3+ or CD8+CD3+ T lymphocytes specifically expressing IFN-gamma in responses to peptide stimulation was analyzed by flow cytometry.
